# Severe Hypotension, Bradycardia and Asystole after Sugammadex Administration in an Elderly Patient

**DOI:** 10.3390/medicina57010079

**Published:** 2021-01-19

**Authors:** Carmen Fierro, Alessandro Medoro, Donatella Mignogna, Carola Porcile, Silvia Ciampi, Emanuele Foderà, Romeo Flocco, Claudio Russo, Gennaro Martucci

**Affiliations:** 1 Department of Medicine and Health Sciences, University of Molise, 86100 Campobasso, Italy; c.fierro@studenti.unimol.it (C.F.); alessandro.medoro@unimol.it (A.M.); donatella.mignogna@studenti.unimol.it (D.M.); carola.porcile@unimol.it (C.P.); s.ciampi@studenti.unimol.it (S.C.); e.fodera@studenti.unimol.it (E.F.); 2 Anesthesia and Intensive Care Unit, “A. Cardarelli” Hospital, Azienda Sanitaria Regionale del Molise, 86100 Campobasso, Italy; romeoflocco@hotmail.com (R.F.); gennaromart@libero.it (G.M.); 3 UOC Governance del Farmaco, Azienda Sanitaria Regionale del Molise, 86100 Campobasso, Italy

**Keywords:** Sugammadex, bradycardia, hypotension, asystole, cardiac arrest, adverse drug reaction

## Abstract

*Background and Objectives:* Sugammadex is a modified γ-cyclodextrin largely used to prevent postoperative residual neuromuscular blockade induced by neuromuscular aminosteroid blocking agents. Although Sugammadex is considered more efficacious and safer than other drugs, such as Neostigmine, significant and serious complications after its administration, such as hypersensitivity, anaphylaxis and, more recently, severe cardiac events, are reported. *Case presentation:* In this report, we describe the case of an 80-year-old male with no medical history of cardiovascular disease who was scheduled for percutaneous nephrolithotripsy under general anesthesia. The intraoperative course was uneventful; however, the patient developed a rapid and severe hypotension, asystole and cardiac arrest after Sugammadex administration. Spontaneous cardiac activity and hemodynamic stability was restored with pharmacological therapy and chest compression. The patient was stabilized and discharged uneventfully on postoperative day 10. *Conclusions:* The potential causes of cardiac arrest after Sugammadex administration have been carefully considered, yet all indications point to Sugammadex as the direct causative agent. On the basis of laboratory and clinical tests, we can exclude among the cause of bradycardia, Kounis syndrome, acute myocardial infarction, coronary spasm and other arrhythmias, but not anaphylaxis. Although Sugammadex is considered an increasingly important option in the prevention of postoperative residual neuromuscular blockade, anesthesiologists should consider it a causative agent of cardiac arrest during surgery. This case highlights the necessity of increased pharmacovigilance and further studies to examine Sugammadex safety and mechanism through which it may cause severe bradycardia, hypotension and cardiac arrest.

## 1. Introduction

The modified γ-cyclodextrin Sugammadex is widely used to antagonize the post-operative residual neuromuscular blockade induced by neuromuscular aminosteroid blocking agents (NMBAs), such as Rocuronium bromide or Vecuronium bromide, used in adult anesthesia to ease endotracheal intubation, mechanical ventilation and surgical access. In detail, Sugammadex binds NMBA clearing nicotinic receptors, quickly reversing residual neuromuscular blockade and reducing the risk of postoperative respiratory complications.

Sugammadex has been reported to be more efficacious and safer than other drugs, such as Neostigmine, with lower incidence of common adverse drug reactions (ADRs) such as postoperative nausea and vomiting, dry mouth, tachycardia and dizziness [1,2,3]. However, the incidence of significant ADRs is almost similar between Sugammadex and Neostigmine: hypersensitivity, anaphylaxis [4,5] and, more recently, severe cardiac events such as coronary vasospasm and acute coronary syndrome [6,7,8], AV block [9], hypotension [10] and bradycardia with or without cardiac arrest [11,12,13,14] are described. 

## 2. Case Presentation

In this report, we describe the case of bradycardia and cardiac arrest after Sugammadex administration in an elderly patient undergoing percutaneous nephrolithotripsy under general anesthesia for renal pelvis and calyceal lithiasis. The patient is an 80-year-old male (158 cm, 55 kg) in polytherapy for hypothyroidism, hyperuricemia, diabetes and chronic obstructive pulmonary disease with 50 mg Levothyroxine, 300 mg Allopurinol, 500 mg Metformin and 322 µg Aclidinium Bromide inhalation, respectively. However, he had no specific past medical history about coronary heart disease or any other cardiovascular disease. The results of preoperative electrocardiogram (ECG), chest X-ray and laboratory tests were normal. The patient went into surgery after premedication with 2 mg Midazolam intravenously (i.v.). Antibiotic prophylaxis was performed with 1 g Cefotaxime i.v.; ECG, noninvasive blood pressure (NIBP), end-tidal carbon dioxide (EtCO_2_) and oxygen saturation (SpO_2_) were monitored throughout the surgery. 

The patient’s initial vital signs were: NIBP 120/70 mmHg, SpO_2_ 98% and heart rate 75 beats/min. General mask ventilation was applied with 10% oxygen, and tracheal intubation was done without accident 2 min after Rocuronium administration. General anesthesia was induced with 140 mg Propofol and a total of 30 mg Rocuronium and, after tracheal intubation, was maintained with 2% (*v*/*v*) Sevoflurane and 0.3 mg Fentanyl. The second part of the surgery was done in a prone position. The patient’s intraoperative vital signs were maintained within the following ranges: systolic blood pressure: 120–130 mmHg; diastolic blood pressure: 70–80 mmHg, heart rate: 80–110 bpm, SpO2: 100%, EtCO2: 37%. An additional 10 mg Rocuronium was administered during the surgery to maintain muscle relaxation, so the total dose of Rocuronium was 40 mg. The total fluid input was 2500 mL (crystalloids). The intraoperative course was uneventful. 

At the surgery end, the Sevoflurane administration was stopped and, after 5 min, 200 mg Sugammadex was administered to the patient: one minute later he developed severe bradycardia with heart rate below 35 beats/min and systolic blood pressure decreased to below 50 mmHg, and was promptly treated with a total of 10 mg Ephedrine and 1 mg Atropine i.v. to restore normal heart rate and systolic blood pressure. However, the patient’s clinical condition rapidly worsened with the onset of severe hypotension, asystole and cardiac arrest. Concomitant cardiopulmonary resuscitation with chest compression was performed for 1 min, restoring spontaneous cardiac activity and hemodynamic stability; the patient was transferred to the intensive care unit (ICU). Arterial blood gas after resuscitation showed: pH 7.32, PaO_2_: 126 mmHg, PaCO_2_: 25 mmHg, base excess: −8.4 mmol/L, HCO_3_: 19 mmol/L, lactate: 8 mmol/L, Na^+^: 136 mEq/L, Mg^2+^: 1.58 mg/dl, K^+^: 3.1 mEq/L, Ca^2+^: 7 mEq/L, glucose: 150 mg/dl and hemoglobin: 10.6 g/dl. Cardiac enzymes and troponins were normal, and postoperative cardiac workup including ECG and transthoracic echocardiography did not show any pathological sign. The patient was stabilized with optimal oxygen saturation level and spontaneous respiration during the following 3 days in ICU, transferred to the urology ward on postoperative day 3 and discharged uneventfully on postoperative day 10. 

## 3. Discussion

The main clues point to Sugammadex as the cause of bradycardia, hypotension and cardiac arrest, both for the temporal proximity of administration and for the occurrence of similar, albeit rare, reports in the literature [15,16] as well as for the drug therapy used to resuscitate the patient based uniquely to Ephedrine and Atropine. Using the Naranjo nomogram, a 7 point-score (probable) was set to this report (Table 1). 

The Sugammadex data sheet clearly states that “Cases of marked bradycardia, some of which have resulted in cardiac arrest, have been observed within minutes after the administration of [Sugammadex]”. The incidence of marked bradycardia at three different Sugammadex doses (2, 4 and 16 mg/kg) in pooled phase 1–3 patients was respectively 1, 1 and 5%. Although, our patient received a dose closer to 4 mg/kg, according to the Sugammadex prescribing information, this dose should not have conferred higher risk for the ADR observed, considering that low-dose Sugammadex (2 mg/kg) does not seem to protect against the chance that life-threatening bradycardia can occur [15]. Moreover, from 2009 to 2020, 292 cases of major cardiac events were reported after Sugammadex/Sugammadex sodium administration in the FAERS database [16], including bradycardia (*n* = 159), cardiac arrest (*n* = 115) and cardio-respiratory arrest (*n* = 18). In the same timeframe, Neostigmine/Neostigmine bromide or methylsulfate has been associated with 75 events, including bradycardia (*n* = 39), cardiac arrest (*n* = 28) and cardio-respiratory arrest (*n* = 8). The analysis, in the same period, of the frequency of total cardiovascular ADRs in respect to allergic or immune-based events shows very close values (567 vs. 574 ADRs, respectively).

Although Sugammadex-induced anaphylaxis is commonly associated with generalized skin rash, wheezing, bronchospasm and tachycardia [4,5] and our patient did not show these clinical signs, allergic-reaction symptoms during anesthesia could be nonspecific, and anaphylaxis-induced cardiovascular collapse has been reported. No other symptoms commonly linked to generalized anaphylaxis (e.g., increased peak inspiratory pressures seen with mechanical ventilation, initial drop in EtCO_2_ and facial or soft palate edema) were observed in the patient. Considering the absence of information about tryptase level or subsequent allergy testing with Sugammadex, we formally cannot exclude anaphylaxis as the primary cause of cardiac arrest [18]. Nonetheless, the fact that our patient responded to a single dose of Ephedrine and Atropine without a relapse of hemodynamic instability and without having to recourse instead to Epinephrine boluses or drip would suggest a greater likelihood of a direct cardiovascular effect rather than secondary to anaphylaxis. Moreover, in the Summary of Product Information released by EMA [19] it is stated that bradycardia induced by Sugammadex should be treated by an anticholinergic agent such as Atropine.

Conversely, no evidence of Kounis syndrome, as recently reported associated with Sugammadex administration, or acute myocardial infarction accompanied by coronary spasm or other arrhythmias were observed [6,7,8], considering the rapid recovery without a relapse of hemodynamic instability, the unnecessity of vasodilators administration and clinical and laboratory normal results during the hours following the cardiac arrest. Moreover, the patient, despite his advanced age, had no medical history of cardiovascular disease or of allergic reactions before this event, unlike patients described in similar reports that are characterized, at least, by hypertension [12,13,14]. Currently, there is no information in the literature about possible drug–drug interaction between Sugammadex and the patient’s polytherapy. 

## 4. Conclusions

In conclusion, although Sugammadex provides an important option for anesthesiologists in the prevention of postoperative residual neuromuscular blockade, it should be considered as a causative agent of cardiac arrest during surgery, directly or following anaphylaxis, even at the lowest recommended doses. For this reason, Sugammadex should be administered slowly, always with full ECG and hemodynamic changes monitoring after its administration [18], and anesthesiologists should be more rigorous in their ADRs reporting to pharmacovigilance agencies. Furthermore, it would be desirable to investigate the genetic and molecular mechanisms that induce the cardiovascular effects of Sugammadex in a specific subpopulation of patients.

## Figures and Tables

**Table 1:** Naranjo nomogram for the assessment of adverse drug reaction (ADR). This questionnaire designed by Naranjo et al. [17] establishes whether an ADR was caused or not by a drug. The ADR is assigned to a probability category from the total score as follows: “definite” if the overall score is 9 or higher, “probable” for a score of 5–8, “possible” for a score of 1–4 and “doubtful” if the score is 0 or less. Bolded numbers apply to the patient’s case.

Assessment Questions	Yes	No	Don’t Know
1. Are there previous conclusive reports on the ADR?	**+1**	0	0
2. Did ADR appear after the suspected drug was given?	**+2**	−1	0
3. Did the ADR improve when the drug was discontinued, or a specific antagonist was given?	**+1**	0	0
4. Did the ADR appear when the drug was re-administered?	+2	−1	**0**
5. Are there alternative causes that could have caused the ADR?	−1	**+2**	0
6. Did the reaction reappear when a placebo was given?	−1	+1	**0**
7. Was the drug detected in any body fluid in toxic concentrations?	+1	0	**0**
8. Was the reaction more severe when the dose was increased, or less severe when the dose was decreased?	+1	0	**0**
9. Did the patient have a similar reaction to the same or similar drugs in any previous exposure?	+1	**0**	0
10. Was the ADR confirmed by any objective evidence?	**+1**	0	0
Total Score		7 = Probable	

## Data Availability

Data and material are available on reasonable request.

## References

[1] Hristovska A.-M., Duch P., Allingstrup M., Afshari A. (2017). Efficacy and safety of sugammadex versus neostigmine in reversing neuromuscular blockade in adults. Cochrane Database Syst. Rev..

[2] Kaufhold N., Schaller S.J., Stäuble C., Baumüller E., Ulm K., Blobner M., Fink H. (2016). Sugammadex and neostigmine dose-finding study for reversal of residual neuromuscular block at a train-of-four ratio of 0.2 (SUNDRO20). Br. J. Anaesth..

[3] Kim Y.-H. (2016). Sugammadex: Watch out for new side effects. Korean J. Anesthesiol..

[4] Miyazaki Y., Sunaga H., Kida K., Hobo S., Inoue N., Muto M., Uezono S. (2018). Incidence of anaphylaxis associated with Sugammadex. Anesth. Analg..

[5] Obara S., Kurosawa S., Honda J., Oishi R., Iseki Y., Murakawa M. (2018). Cardiac arrest following anaphylaxis induced by sugammadex in a regional hospital. J. Clin. Anesth..

[6] Hoshino K., Kato R., Nagasawa S., Kozu M., Morimoto Y. (2015). A case of repetitive cardiac arrest due to coronary vasospasm after Sugammadex administration. Masui. Jpn. J. Anesthesiol..

[7] Ko M.J., Kim Y.H., Kang E., Lee B.C., Lee S., Jung J.W. (2016). Cardiac arrest after sugammadex administration in a patient with variant angina: A case report. Korean J. Anesthesiol..

[8] Yanai M., Ariyoshi K. (2020). Two Cardiac Arrests that Occurred after the Administration of Sugammadex: A Case of Kounis Syndrome. Case Rep. Emerg. Med..

[9] Saito I., Osaka Y., Shimada M. (2015). Transient third-degree AV block following sugammadex. J. Anesth..

[10] Kokki M., Ali M., Turunen M., Kokki H. (2012). Suspected unexpected adverse effect of sugammadex: Hypotension. Eur. J. Clin. Pharmacol..

[11] Bilgi M., Demirhan A., Akkaya A., Tekelioglu Y., Kocoglu H. (2014). Sugammadex associated Persistent Bradycardia. Int. J. Med. Sci. Public Health.

[12] Sanoja I.A., Toth K.S. (2019). Profound Bradycardia and Cardiac Arrest After Sugammadex Administration in a Previously Healthy Patient: A Case Report. A A Pract..

[13] Oliveira C., Marques C., Simões V., Spencer L., Poeira R., Casteleira M. (2019). Severe bradycardia and asystole associated with sugammadex: Case report. Braz. J. Anesthesiol..

[14] Bhavani S.S. (2018). Severe bradycardia and asystole after sugammadex. Br. J. Anaesth..

[15] BRIDION: Highlights of Prescribing Information. https://www.merck.com/product/usa/pi_circulars/b/bridion/bridion_pi.pdf.

[16] FDA Adverse Events Reporting System (FAERS) Public Dashboard. https://fis.fda.gov/sense/app/d10be6bb-494e-4cd2-82e4-0135608ddc13/sheet/45beeb74-30ab-46be-8267-5756582633b4/state/analysis.

[17] Naranjo C.A., Busto U., Sellers E.M., Sandor P., Ruiz I., A Roberts E., Janecek E., Domecq C., Greenblatt D.J. (1981). A method for estimating the probability of adverse drug reactions. Clin. Pharmacol. Ther..

[18] Hunter J.M., Naguib M. (2018). Sugammadex-induced bradycardia and asystole: How great is the risk?. Br. J. Anaesth..

[19] BRIDION—Summary of Product Information Released by EMA. https://www.ema.europa.eu/en/documents/product-information/bridion-epar-product-information_en.pdf.

